# Microvascular decompression for trigeminal neuralgia caused by persistent trigeminal artery associated with craniosynostosis: a case report

**DOI:** 10.1186/s13256-022-03490-9

**Published:** 2022-07-29

**Authors:** Tao Sun, Qinghao Huang, Chuangfeng Li, Wentao Wang, Longshuang He, Jinlong Liu, Chao Yang

**Affiliations:** 1 Department of Neurosurgery, The First Affiliated Hospital of Sun Yat-Sen University, No 58th, Zhongshan Er Road, Guangzhou, 510080 China; 2 Center of Universal Medical Imaging Diagnostic, No 80th, Zhongshan Er Road, Yuexiu District, Guangzhou, 510080 China; 3 Department of Neurosurgery, The First Affiliated Hospital of Guangdong Pharmaceutical University, No. 19, Nonglinxia Road, Yuexiu District, Guangzhou, 510080 China

**Keywords:** Persistent trigeminal artery, Trigeminal neuralgia, Craniosynostosis, Microvascular decompression

## Abstract

**Background:**

Persistent trigeminal artery (PTA) is a rare arterial anastomosis between the basilar artery (BA) and internal carotid artery (ICA). It plays an indispensable role in a number of neurological disorders, including trigeminal neuralgia (TN).

**Case presentation:**

We report a unique case of a 58-year-old Han female patient with TN caused by PTA associated with craniosynostosis. Preoperative three-dimensional time-of-flight (3D-TOF) magnetic resonance (MR) and 3D constructive inference in steady state (3D-CISS) imaging showed that the PTA run though Meckel’s cave. Complete pain relief was immediately achieved after microvascular decompression (MVD), without facial numbness and other complications. No recurrence was recorded at the 1-year follow up.

**Conclusions:**

Microvascular decompression is a feasible option for the treatment of complex TN combined with other abnormalities. For patients deemed suitable for percutaneous balloon compression, PTA should be ruled out. Preoperative 3D-TOF and 3D-CISS MR imaging were essential to identify PTA neurovascular conflicts.

## Introduction

Persistent trigeminal artery (PTA) is the most common abnormal arterial anastomosis between the basilar artery (BA) and internal carotid artery (ICA) [1, 2], and usually originates from the cavernous segment of the ICA. The incidence of PTA has been reported to be < 1%, and the reported incidence of PTA as culprit vessel of trigeminal neuralgia (TN) is 0.2–0.6% [3–5]. Craniosynostosis is a rare skull malformation that is caused by premature closure of cranial sutures, with an incidence of 1:2000–2500 among newborns [6, 7]. Surgery on such patients is extremely difficult given cerebellopontine angle cistern crowding. We report here the first case of TN caused by compression of the PTA associated with craniosynostosis. Our aim is to share our experience in treating TN caused by PTA.

This study was approved by the Ethics Committee of First Affiliated Hospital of Sun Yat-sen University (Approval Number IIT-2021-378) and has been performed in accordance with the ethical standards laid down in the 1964 Declaration of Helsinki and its amendments. Details that might disclose the ID of the patient were omitted. Written informed consent was obtained from the patient for publication of this case report and any accompanying images.

## Case presentation

**Fig. 1:**
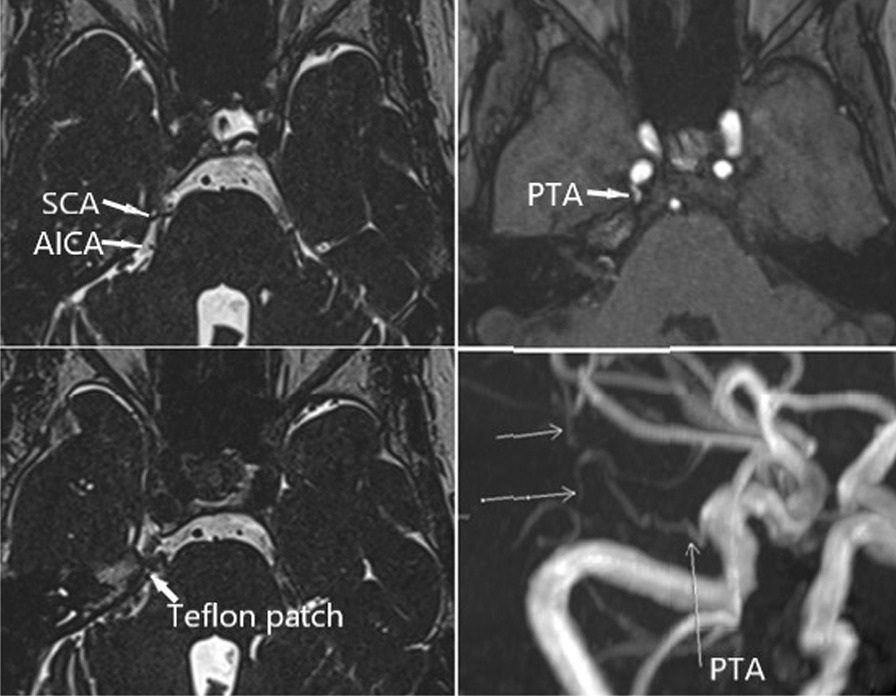
PTA originated from the junction of the C3 and C4 segments of the ICA and went up around the inner margin of Meckel’s cave, eventually terminating into the right SCA which anastomosed with the AICA. *PTA* Persistent trigeminal artery, *ICA* internal carotid artery, *SCA* superior cerebellar artery, *AICA* anterior inferior cerebellar artery

**Fig. 2:**
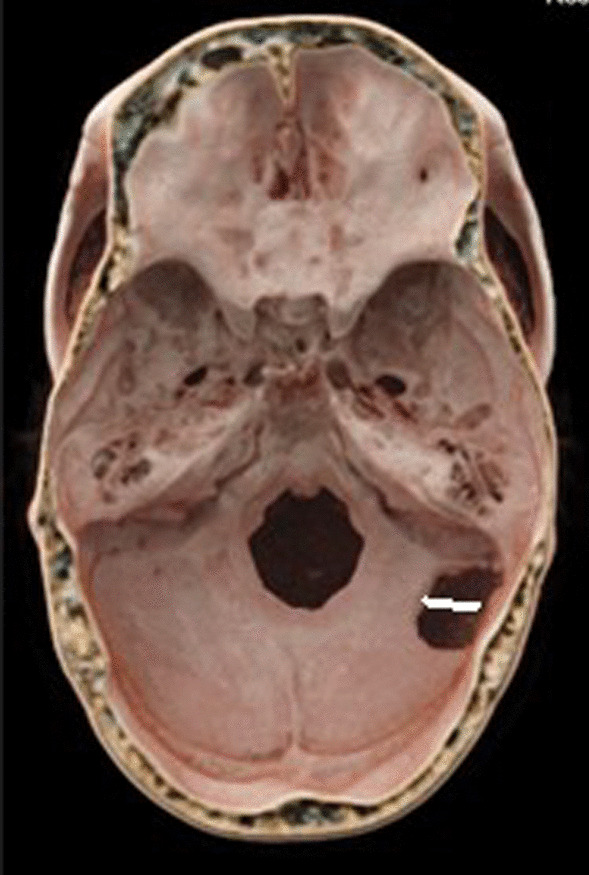
Three-dimensional reconstruction of the patient’s skull shows a narrow skull. The posterior-lateral hole was the bone window of the first MVD in another hospital, and the part in front of the white line was used in the current operation to fully expose the margins of the sigmoid sinus and transverse sinus. *MVD* Microvascular decompression

A 58-year-old Han woman presented to our department in November 2020 complaining of typical TN of the second and third branches of the nerve in the right side of the face for the past 20 years. The pain started during physical activities. She took carbamazepine and had gradually increased the dose to 1200 mg per day, but severe pain remained and the high doses caused severe side effects. TN had been diagnosed 12 years previously at another hospital, where microvascular decompression (MVD) was carried out but failed to exposed the trigeminal nerve. Neurological examinations showed no other abnormalities of the cranial nerve. Three-dimensional time-of-flight (3D-TOF) magnetic resonance (MR) and three-dimensional constructive inference in steady state (3D-CISS) imaging showed obvious neurovascular conflicts (NVCs) due to PTA and the trigeminal nerve and that the PTA originated from the junction of the C3 and C4 segments of the ICA and went up around inner margin of Meckel’s cave, eventually to terminate into the right superior cerebellar artery (SCA) which anastomosed with the anterior inferior cerebellar artery (AICA) (Fig. 1). Following multidisciplinary consultation, we determined that the patient had a narrow head and made a diagnosis of scaphocephaly (Fig. 2).

**Fig. 3:**
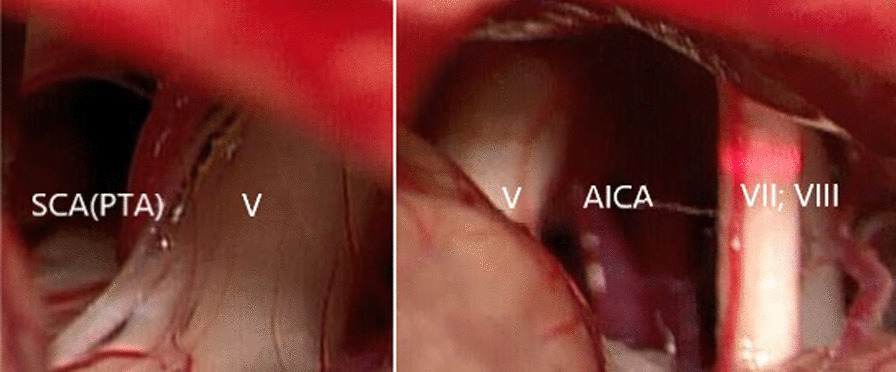
The SCA, which is the terminal branch of the PTA, compresses the trigeminal nerve at Meckel’s cave, and the AICA compresses the ventral lateral and head lateral nerves. *V* Trigeminal nerve, *VII* facial nerve, *VIII* vestibulocochlear nerve; all other abbreviations as defined in Fig. 1 caption

We found complex NVCs intraoperatively. The SCA, which is the terminal branch of PTA, compressed the cisternal segment of the trigeminal nerve, and the AICA compressed the trigeminal nerve at the root entry zone (REZ). Teflon patches were inserted between the trigeminal nerve and vessels deemed to be responsible for the pain, to achieve complete decompression and release of all culprit vessels (Fig. 3). The patient achieved full pain relief after MVD, without facial numbness and other complications. No recurrence was recorded at the 1-year follow-up.

## Discussion

In our case, TN was caused by compression of the PTA, which is an abnormal arterial anastomosis between the ICA and BA. As PTA originates from the ICA, it usually runs medially to the first branch of the trigeminal nerve and extradurally beneath the oculomotor and trochlear nerves, following which it passes the petroclinoidal ligament or perforates the dura around the clivus to join the BA. Only rarely does the PTA enter the posterior cranial fossa, either from Meckel’s cave or from the dural foramen, and cause cerebellum vascularization with no anastomoses with the BA [8]. We suspect the coexistence of craniosynostosis and PTA may complicate the anatomical relationship and increase the possibility of NVCs.

### PTA and TN

During the developmental stage of a 4-mm embryo, the ICA supplies the longitudinal neural arteries via the trigeminal artery, optic artery, hypoglossal artery and proatlantal intersegmental artery. In subsequent developmental stages the arteries normally regress and disappear, but if there is incomplete regression or the ipsilateral posterior cerebral artery does not develop properly, a persisting anastomosis, such as PTA, may occur [9].

Both the REZ and cisternal part of the nerve are reported to be the responsible sites of TN [10]. Chidambaranathan et al. reported that PTA originated from the posterolateral margin of the posterior bend of the cavernous segment of the ICA, ran posterolaterally and inferiorly around the dorsum sellae and communicated with the BA in the prepontine cistern [8]. They also reported that the PTA compressed and distorted the REZ of the trigeminal nerve laterally [8]. In our case, the PTA ran across Meckel’s cave and compressed the cisternal segment of the nerve caudally. de Bondt et al. found that PTA played a vital important role in TN, reporting that the incidence of PTA among patients with TN was 2.2%, which was much higher than that found in the general population [9]. Owing to its abnormal blood pattern, the PTA could be large and potentially tortuous compared to the SCA, which is the most common culprit vessel of TN. Compression of the PTA could lead to chronic compression and microtrauma, resulting in hyperactivity of the nerve and ultimately to TN [10]. Some studies found that branches of the PTA can also be culprit vessels of TN [11–14]. PTA-related aneurysm [15] or arteriovenous malformation (AVM) [16] have also been reported to negatively affect the trigeminal nerve.

Hemorrhage has been reported to be a severe complication in percutaneous balloon compression (PBC) procedure [17]. Also, PBC might injure the PTA in the Meckel’s cave, the balloon catheter could puncture the PTA and/or shearing compression could tear PTA during the formation of “pear head”. Consequently, preoperative 3D-TOF and 3D-CISS MR imaging are essential to identify the PTA to rule out PBC for the patients.

### Craniosynostosis and TN

Exposure of the trigeminal nerve, especially the REZ of the nerve, is largely confined to patients with narrow posterior cranial fossa [18, 19]. However, in our patient, we exposed the trigeminal nerve and achieved complete decompression of the nerve, freeing the patient from pain without any complications. The methods we used included full exposure of the margin of the retrosigmoid and transverse sinus and dynamic retraction of the cerebellum using suction and bipolar. Elevation of the head of the bed and mannitol can be used to decrease intracranial pressure and ease the effects of the operation.

The CT imaging of our patient indicated that the osteoclasia around the foramen oval on the right side does not form a complete bony boundary, thus greatly increasing the possibility of ICA injury during PBC. The incomplete bone boundary might attribute to craniosynostosis.

## Conclusions

We report the first case of TN caused by PTA associated with craniosynostosis. MVD was a feasible option for complex TN combined with other abnormalities. For patients awaiting PBC, PTA should be ruled out. Preoperative 3D-TOF and 3D-CISS MR image were essential to identify PTA NVCs.

## Data Availability

Data of the patient are recorded in the manuscript in detail. For more information, please contact the corresponding author.

## References

[1] Bouthillier A, van Loveren HR, Keller JT. Segments of the internal carotid artery: a new classification. Neurosurgery. 1996;;38(3):425-32; discussion 432-3. 10.1097/00006123-199603000-00001. 8837792

[2] Tulsi RS, Locket NA. Persistent trigeminal artery: an anatomical study. Aust N Z J Surg. 1985;55:397–402. 10.1111/j.1445-2197.1985.tb00908.x. 3870171

[3] Salas E, Ziyal IM, Sekhar LN, Wright DC. Persistent trigeminal artery: an anatomic study. Neurosurgery. 1998;43(3):557-61; discussion 561-2. 10.1097/00006123-199809000-00082. 9733310

[4] Freitas PE, Aquini MG, Chemale I. Persistent primitive trigeminal artery aneurysm. Surg Neurol. 1986;26:373–4. 10.1016/0090-3019(86)90139-4. 3750194

[5] Fields WS. The significance of persistent trigeminal artery. Carotid-basilar anastomosis. Radiology. 1968;91:1095–101. 5699609

[6] Hankinson TC, Fontana EJ, Anderson RC, Feldstein NA. Surgical treatment of single-suture craniosynostosis: an argument for quantitative methods to evaluate cosmetic outcomes. J Neurosurg Pediatr. 2010;6:193–7. 10.3171/2010.5.PEDS09313. 20672943

[7] Persing JA. MOC-PS(SM) CME article: management considerations in the treatment of craniosynostosis. Plast Reconstr Surg. 2008;121:1–11. 10.1097/01.prs.0000305929.40363.bf. 18379381

[8] Chidambaranathan N, Sayeed ZA, Sunder K, Meera K. Persistent trigeminal artery: a rare cause of trigeminal neuralgia—MR imaging. Neurol India. 2006;54:226–7. 16804286

[9] de Bondt BJ, Stokroos R, Casselman J. Persistent trigeminal artery associated with trigeminal neuralgia: hypothesis of neurovascular compression. Neuroradiology. 2007;49:23–6. 10.1007/s00234-006-0150-8. 17089114

[10] Headache Classification Committee of the International Headache Society (IHS). The International Classification of Headache Disorders, 3rd edition. Cephalalgia. 2018;38:1–211. 10.1177/0333102417738202. 29368949

[11] Ling MM, Gupta M, Acharya J. Trigeminal neuralgia associated with a variant of persistent trigeminal artery. Radiol Case Rep. 2020;15:2225–8. 10.1016/j.radcr.2020.08.057. PMC7486686 32952760

[12] Kato N, Tanaka T, Sakamoto H, Arai T, Hasegawa Y, Abe T. Identification of a persistent primitive trigeminal artery following the transposition technique for trigeminal neuralgia: a case report. Pain Res Manag. 2011;16:357–9. 10.1155/2011/987865. PMC3206786 22059208

[13] Lee SH, Koh JS, Lee CY. Trigeminal neuralgia caused by an anomalous posterior inferior cerebellar artery from the primitive trigeminal artery: case report. Cerebellum. 2011;10:199–203. 10.1007/s12311-011-0249-2. 21279490

[14] Yamada Y, Kondo A, Tanabe H. Trigeminal neuralgia associated with an anomalous artery originating from the persistent primitive trigeminal artery. Neurol Med Chir (Tokyo). 2006;46:194–7. 10.2176/nmc.46.194. 16636511

[15] Chen WH, Tsai TH, Shen SC, Shen CC, Tsuei YS. A case of giant thrombosed persistent primitive trigeminal artery aneurysm presenting with trigeminal neuralgia and successfully treated by a covered stent: case report and review of literature. Clin Neuroradiol. 2015;25:207–10. 10.1007/s00062-014-0314-6. 24867146

[16] Choudhri O, Heit JJ, Feroze AH, Chang SD, Dodd RL, Steinberg GK. Persistent trigeminal artery supply to an intrinsic trigeminal nerve arteriovenous malformation: a rare cause of trigeminal neuralgia. J Clin Neurosci. 2015;22:409–12. 10.1016/j.jocn.2014.06.007. 25070632

[17] Gatto L, Tacla R, Koppe GL, Junior ZD. Carotid cavernous fistula after percutaneous balloon compression for trigeminal neuralgia: endovascular treatment with coils. Surg Neurol Int. 2017;8:36. 10.4103/sni.sni_443_16. PMC5369261 28458950

[18] Hardaway FA, Holste K, Ozturk G, Pettersson D, Pollock JM, Burchiel KJ, *et al*. Sex-dependent posterior fossa anatomical differences in trigeminal neuralgia patients with and without neurovascular compression: a volumetric MRI age- and sex-matched case-control study. J Neurosurg. 2019;132:631–8. 10.3171/2018.9.JNS181768. 30717058

[19] Cheng J, Fang Y, Zhang H, *et al*. Quantitative study of posterior fossa crowdedness in hemifacial spasm. World Neurosurg. 2015;84:920–6. 10.1016/j.wneu.2015.04.040. 26341431

